# An Indoor Obstacle Detection System Using Depth Information and Region Growth

**DOI:** 10.3390/s151027116

**Published:** 2015-10-23

**Authors:** Hsieh-Chang Huang, Ching-Tang Hsieh, Cheng-Hsiang Yeh

**Affiliations:** 1 Department of Information Technology, Lee-Ming Institute of Technology, New Taipei City 24346, Taiwan; E-Mail: sanmic@mail.lit.edu.tw; 2 Department of Electrical Engineering, Tamkang University, New Taipei City 25137, Taiwan; E-Mail: porkp617@hotmail.com

**Keywords:** obstacle detection, Kinect, depth map, travel aid

## Abstract

This study proposes an obstacle detection method that uses depth information to allow the visually impaired to avoid obstacles when they move in an unfamiliar environment. The system is composed of three parts: scene detection, obstacle detection and a vocal announcement. This study proposes a new method to remove the ground plane that overcomes the over-segmentation problem. This system addresses the over-segmentation problem by removing the edge and the initial seed position problem for the region growth method using the Connected Component Method (CCM). This system can detect static and dynamic obstacles. The system is simple, robust and efficient. The experimental results show that the proposed system is both robust and convenient.

## 1. Introduction

According to new statistics [1], there are 285 million visually impaired people relying on the guide cane or guide dogs to move around freely in the world. However, not every visually impaired person can easily pair successfully with guide dogs and there is often a long wait for an animal.

Most visually impaired people use a cane to touch an obstacle, to assess the position of the obstacle and avoid it. Sometimes at the point when they touch the obstacle, the danger is unavoidable. These two methods for travel are neither convenient nor safe. Using computer vision technology reduces this problem. The efficient detection of obstacles is important. In recent years, there have been many developments in computer vision for this field. Many studies have proposed obstacle detection methods. In [2] Obstacle detection can be classified into three categories: Electronic travel aids (ETAs), electronic orientation aids (EOAs) and position locator devices (PLDs). However, this paper classifies obstacle detection into three categories. One uses non-depth information, a second uses depth information and the third uses neither.

There are many proposed methods for the first category, such as [3,4,5,6,7,8]. Ma *et al.* [3] proposed an object detection algorithm that uses edges and motion. The motion-information is used to determine the dynamic obstacles and the edge-information is used to determine obstacles. This information is combined with free space detection to determine the position of the obstacles. Zhang *et al.* [4] proposed an obstacle detection algorithm that uses a single camera. This uses edge detection to segment objects. However, these methods require a simple texture for the surface of the ground. Chen *et al.* [5] proposed an obstacle detection method that uses a saliency map. This uses a threshold value to determine the position of the obstacles. However, this method requires that there are few obstacles in the execution environment. Ying *et al.* [6] proposed an obstacle detection method that uses a gray-scale image. This method searches the region of interest (ROI) in the gray-scale image and then determines the location of obstacles. However, this method uses a gray-scale image, so it is easily affected by illumination. These methods are very robust if there is sufficient light, but not if there is insufficient light. The proposed system uses Kinect directly to capture the depth map, so it addresses these drawbacks.

The second category of methods for obstacle detection is been proposed in [9,10,11,12,13,14,15,16,17,18,19,20,21,22,23,24]. These methods detect obstacles using depth information. This is obtained from various capture devices, such as stereovision cameras, Leap Motion controllers [25], laser rangefinders [26], RealSense 3D Cameras [27] or Kinect sensors. Zollner *et al.* [8] just given a proof-of-concept idea of a mobile navigational aid, but the implementation of the proposed Kinect application was lacked. Filipe *et al.* [10] applied Neural Network to extract the features from the depth information captured by Kinect sensor and the extracted features are enabled to detect possible obstacles. In general, depth information of obstacles is really similar to the surrounding floor (ground plane) and the trained NN may be hard to separate the obstacles from the floor. Hotaka *et al.* [11] proposed Kinect cane system and tactile inform system, that is different from ours. Above three papers don’t remove ground plane from depth map. However, our proposed system resolves the over-segmentation problem by removing the edge and the initialize seed position problem for the growth method (RGM) using the Connected Component Method (CCM). The RGM concept is simple. We only need a certain numbers of seed point to represent the property we want, then grow the region. The vocal inform system of our proposed system is more intuitive. And we do not change cane of visually impaired people. Zhang *et al.* [12] proposed an obstacle detection algorithm that uses a U-V disparity map analysis. This combines straight-line fitting and the standard Hough Transform [28] to determine the location of obstacles. However, the U-V disparity map is generated using two webcams, so the degree of illumination affects the performance of the system. In [13], Gao *et al.* use a 3D camera to obtain the depth map. This study combines straight-line fitting, the standard Hough Transform and a U-V disparity map to determine the location of obstacles. Choi *et al.* [14] used a Kinect sensor to obtain color images and depth maps (RGB-D images). This study uses edge detection for both color images and depth maps and then processes these edge images by morphology [29]. The results for the two images are then combined to determine the position of obstacles. However, the color image used in this study is still affected by illumination and the ground plane affects obstacle detection. The proposed system addresses these two problems.

For the third category of systems for obstacle detection, Brock *et al.* [30] used a vibrotactile belt to convey the position and distance to an obstacle using the position and strength of the vibrations. For more detail about a vibrotactile belt, please refer to [31]. The vOICe’s Glasses for the Blind [32] are a wearable device that is equipped with a webcam and translates video data into a sound stream. Mann *et al.* [33] presented a novel head-mounted navigational aid that uses Kinect and vibrotactile devices built onto a helmet.

The method detailed in [34] does not process the ground, but segments object directly to calculate the standard deviation using an object’s depth value and then determines whether it is an obstacle using the scale of the object’s standard deviation. Although this detection method is simple, smaller objects on the ground are not detected. The proposed system filters the ground out before obstacle detection is begun, so this issue is eliminated. The system used in [35] is an autonomous navigation system that uses a finite state machine that is taught by an Artificial Neural Network (ANN) in an indoor environment. The system used in [36] uses machine learning for this field. The design goals for the proposed system are cost-efficiency, robustness and convenience. The system must address the ground plane problem, in order to detect rising stairs, descending stairs and static and dynamic obstacles.

The remainder of the paper is organized as follows. Section 2 gives a system overview and the details of the system. Section 3 gives the experimental results for different environments and the experimental results for two blind subjects and ten blindfolded subjects. Finally, a conclusion and details of future work are given in Section 4.

## 2. Proposed Methods

### 2.1. System Architecture

**Figure 1:**
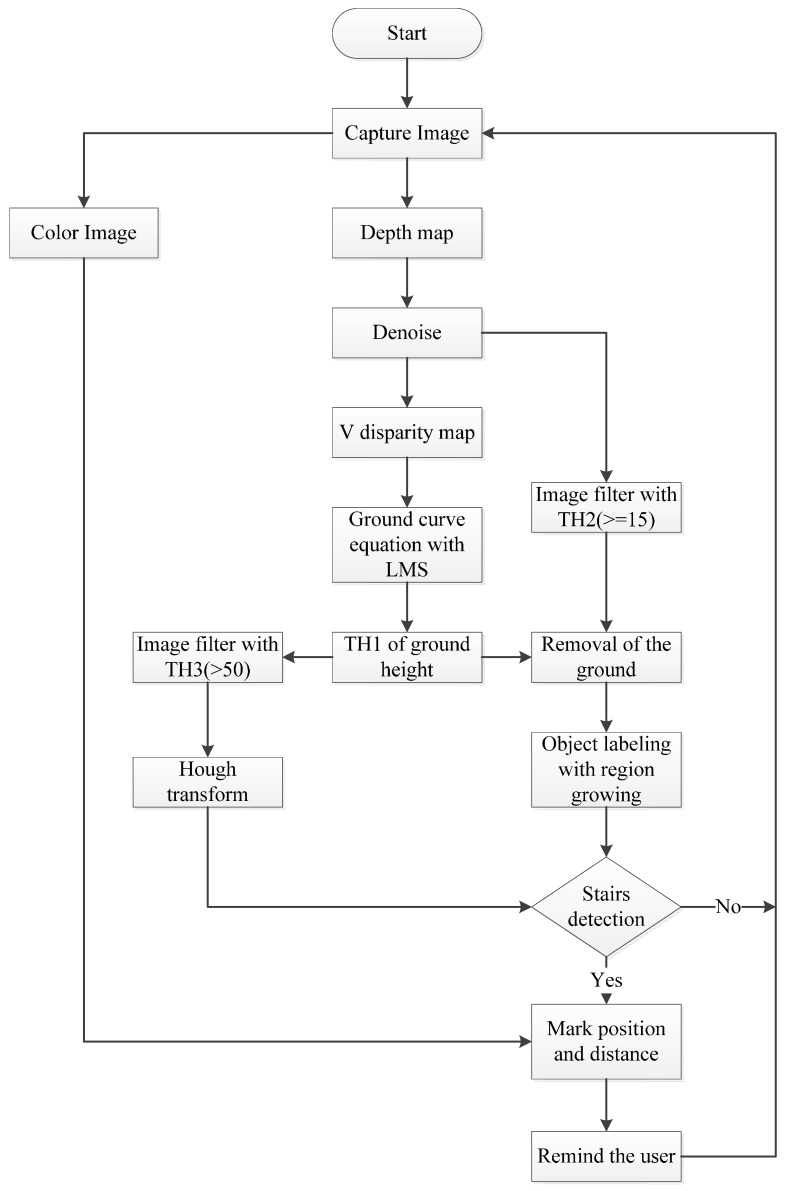
The system flowchart.

The proposed system flowchart is shown in Figure 1. Firstly, the morphology is dilated and eroded to remove the distracting noise of the depth map and the Least Squares Method (LSM) in a quadratic polynomial is used to approximate ground curves and to determine the ground height threshold in the V-disparity. The system then searches for dramatic changes in the depth value, depending on the ground height threshold, to determine stair-edge points. The Hough Transform is then used to determine the location of the drop line [37]. In order to strengthen the characteristics of the different objects and to overcome the drawbacks of the region growth method [38], edge detection is used to remove the edge. The ground height threshold and the features of the ground are then used to remove ground plane. The system then uses the region growth method to label the tags on different objects and analyzes each object to determine whether the object is a stair. Finally, the system allows users to navigate and gives them a vocal message about the distance to the obstacle and the obstacle category using Text To Speech (TTS).

### 2.2. Noise Reduction

**Figure 2:**
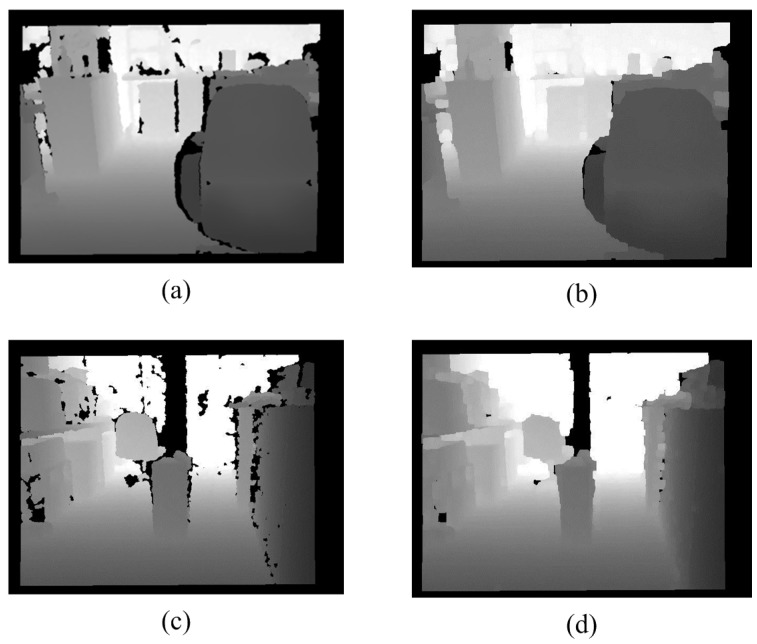
Noise Removal. (**a**) Original depth map; (**b**) Processing result; (**c**) Original depth map; (**d**) Processing result.

Because of the limitations of the Kinect hardware, a depth map can be broken. In order to make the depth map more complete, some simple morphology processing is used. This paper uses a closing operation for morphology to repair the black broken areas. Figure 2 shows that the processed depth maps are better than the original depth maps.

### 2.3. Ground Height Detection

**Figure 3:**
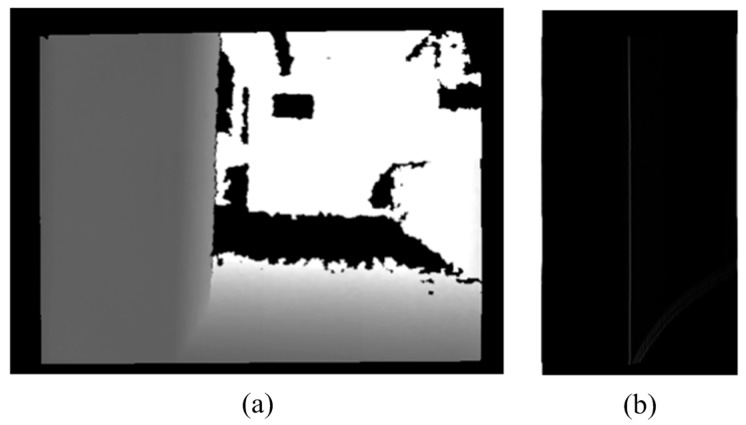
The relationship between the depth map and the V-disparity. (**a**) Depth map; (**b**) V-disparity.

A UV disparity map is composed of the U disparity map and the V disparity map from the depth map. Figure 3 shows that the V-disparity [39] concept simplifies the process of separating obstacles in an image, where “V” corresponds to the vertical coordinate in the (u, v) image coordinate system. Similarly, the U-disparity concept simplifies the process of separating obstacles in an image, where “U” corresponds to the vertical coordinate in the (u, v) image coordinate system.

**Figure 4:**
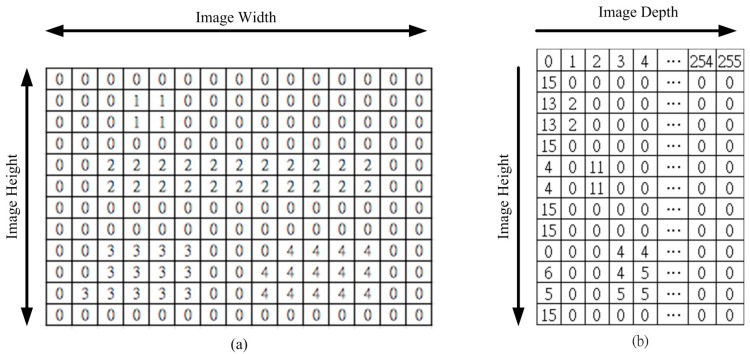
A schematic diagram of the V disparity map. (**a**) Depth map; (**b**) V disparity map.

A UV disparity map [40] is a statistical method that is similar to a histogram. However, the statistical target is different. The proposed system only uses V-Disparity because the effect is better. Figure 4a shows that this table is a depth map. The statistics for different depth values are gathered, row-by-row, and the results are shown in Figure 4b. For example, there are 15 zeros in row one in Figure 4a, so the position of Row 2 and Column 1 in Figure 4b records this value (15). This means that the depth value, 0, has an image height of 15.

**Figure 5:**
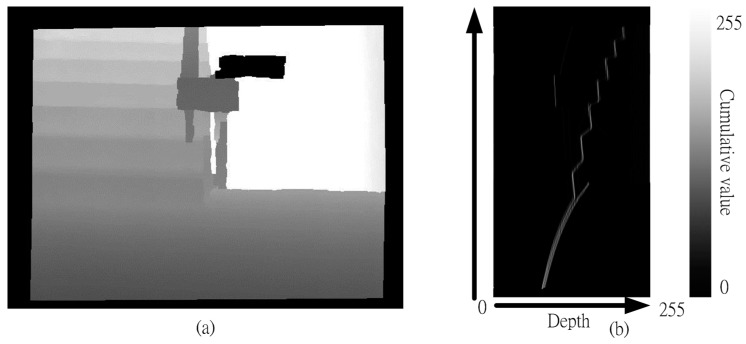
(**a**) The depth map with noise removed and (**b**) the V disparity map image.

The detection needs for subsequent steps require that noise must be removed from the captured depth map this must be projected into the V disparity map, as shown in Figure 5. The Y-axis height of the V disparity map corresponds to the Y-axis height of the depth maps, as shown in Figure 5, so the vertical length of an image represents the height of the actual object in the image. If the object is closer to the right side of the depth map, the distance between the object and the sensor is greater. The greater the pixel value in the V disparity map, the bigger is the object in the image. The normalization equation for the cumulative amount of depth is shown in the following equation. The cumulative value must be between 0 and 255. The cumulative value is statistical value of depth value of the row of the V disparity map image, and the Max cumulative value is image wide value of the depth map:

(1) $$Depth\;cumulative\;value=\frac{cumulative\;value}{Max\;cumulative\;value}*255$$

**Figure 6:**
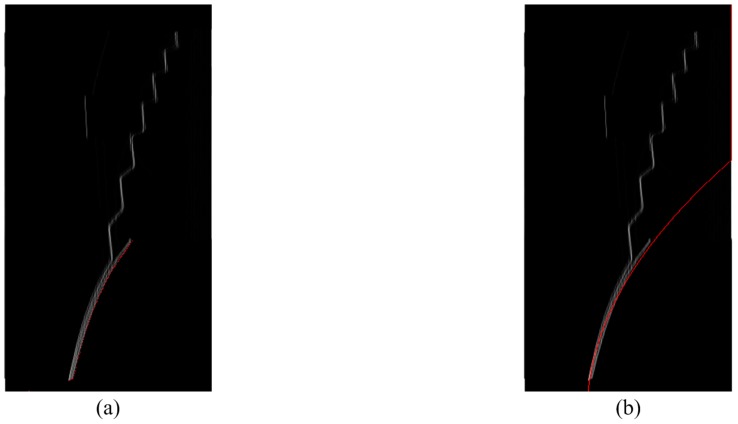
The ground curve in V disparity map. (**a**) Segment consisting of points (red); (**b**) The line of the equation (red).

**Figure 7:**
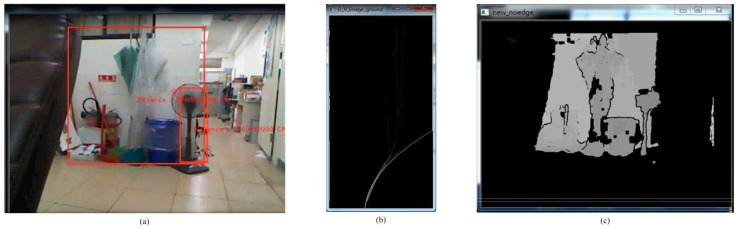
The scene without people. (**a**) Real scene; (**b**) V-disparity; (**c**) Depth map.

**Figure 8:**
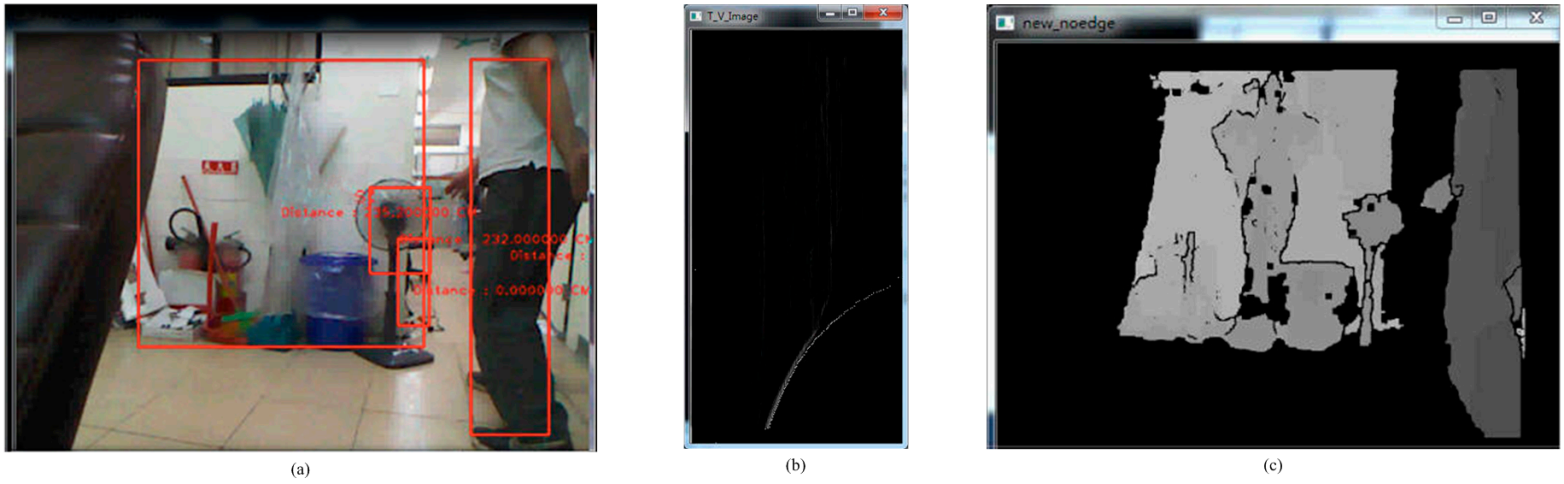
The scene with people. (**a**) Real scene; (**b**) V-disparity; (**c**) Depth map.

**Figure 9:**
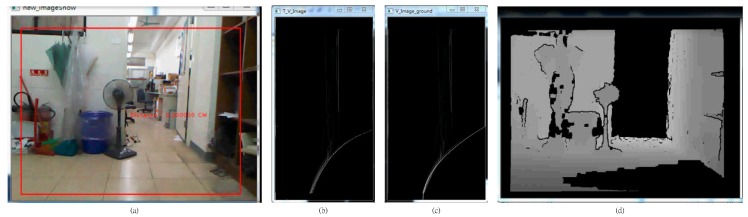
No offset. (**a**) Real scene; (**b**) No LSM Curve; (**c**) LSM Curve without offset; (**d**) Depth map.

**Figure 10:**
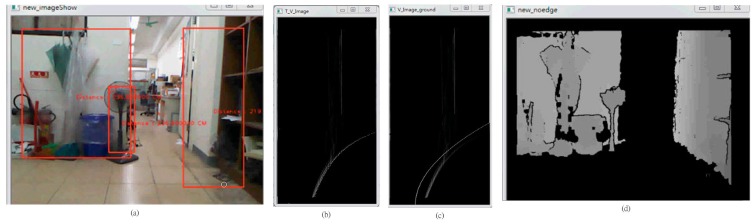
Offset value = 20. (**a**) Real scene; (**b**) No LSM Curve; (**c**) LSM Curve without offset; (**d**) Depth map.

**Figure 11:**
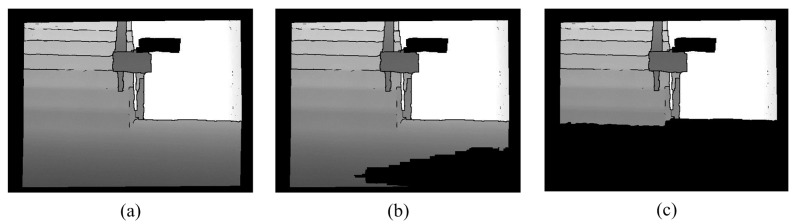
The result of the offset. (**a**) Original depth map; (**b**) Result image before offset; (**c**) Result image after offset.

According to [11], the ground is a rising curve in a V disparity map. The LSM is used to determine the equation of the curve, as shown in Figure 6 and Equation (2).

(2) $$ay^{2}+by+c=d$$

where a, b and c respectively represent the parameters of the equation, y is the image height and d is the horizontal axis value (0 to 255) in the V-disparity map. However, we want to find out a quadratic equation to closer ground curve strip, then use it to remove ground plane. The ground plane is not only a simple line in the V-disparity map. Because pixels that are the same height in a depth map can have a different depth value, the curve becomes a strip, so several approximation targets, such as the minimum, the maximum, the mean and the specific value of every row of V-disparity map are used (the rightmost value of the strip, the leftmost value of the strip, the middle value of the strip on x-axis).When the obstacle is on the ground, these methods do not work. To address this problem, the proposed method uses the quadratic offset equation, which is shown as Equation (3):

(3) $$TH1=ay^{2}+by+c-offset=d-offset$$

where *TH*1 is the shifted threshold depending on the ground height. The ground height threshold value indicates a height in the depth map and the minimum value cannot be less than *TH*1. The appropriate offset value is 35, which is obtained through experience. The offset value affects the removal of the ground, so several offset values, such as the minimum, the maximum, the mean and the specific value, are tried. The offset value controls the location of the approximation curve for the disparity map. The quadratic offset equation is the fastest and simplest method. Comparing the disparity map in Figure 7 with that in Figure 8, it is seen that the depth value of the ground plane (background) is greater than the depth value of the obstacle (foreground) for the same height. Figure 9 shows that the mean method (no offset) does not completely remove the ground plane. Therefore, the maximum method does not remove the ground plane either. In contrast, the minimum method is perhaps the best, but the depth of the obstacle interferes with this method. Because the depth value for the background is greater than the depth value for the foreground for the same height in the V-disparity, the minimum method cannot be used directly. Using the LSM to subtract the specific value is the best method, as shown in Figure 10. Figure 11 shows that Equation (3) improves the robustness of the system.

### 2.4. Removal of the Edge

In the depth map, the depth represents the distance between the objects and the sensor. The variation in depth demonstrates whether the obstacles are the same. Variations in depth are usually not too significant for a specific object. If there are different objects, the relationship between the distances causes a significant variation in the depth. In this paper, in order to clarify the characteristics of different objects, the strong edge is removed. There are many edge detection methods, such as Roberts, Prewitt, Sobel, Laplace and Canny. In this paper, a function to detect the edge uses the following Equation (4):

(4) $$P(x,y)=\left\{ \begin{array}{l} 0\;,\;if\;\sum_{x_{n},y_{n}\in S_{n}}\left|P(x_{n},y_{n})-P(x,y)\right|\;\geq TH2\\ unchange\;,\;others \end{array}\right.$$

**Figure 12:**
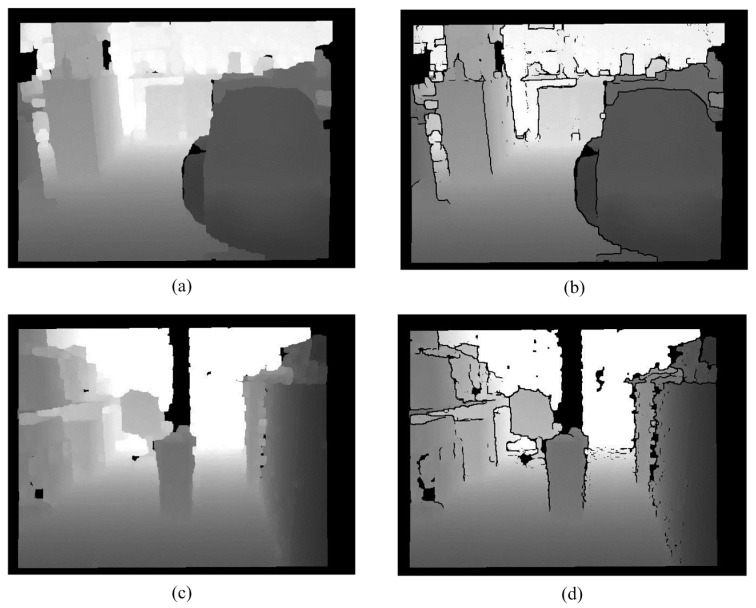
Removal of the edge. (**a**) Noiseless image; (**b**) Processing result; (**c**) Noiseless image; (**d**) Processing result.

The processing result is shown in Figure 12. Here, P(⋅) represents the pixel value of the coordinates (x,y) and TH2 represents the threshold. If $P(x_{n},y_{n})$ is P(x,y)'s neighboring pixel and $S_{n}$ is a set of P(x,y)'s neighboring pixels and the image is traversed using Equation (4), then the edges in the image can be detected. When all of the edges in the depth map are found, objects can be isolated, so segmentation is accurate.

### 2.5. The Detection of Descending Stairs

**Figure 13:**
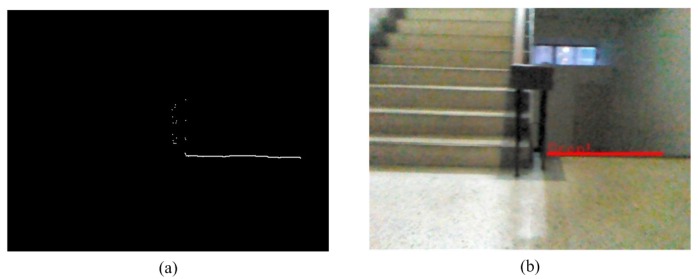
The results for the detection of descending stairs. (**a**) Suspicious points of downstairs depth map; (**b**) The results of the Hough transform.

In this section, a method to search and record points that exhibit significant variation from the noiseless image is proposed. In this study, the pixel values are larger than the setting threshold (50) and are defined as significant variation. The ground height threshold (*TH*3) is then used to filter out possible points, as shown in Figure 13a. These depth values of vertical adjacent point are very difference. After filtering, they become a group of points. We call these points “possible points”. In depth map, the Hough Transform technique transforms the possible points into edge line of descending stairs. The Hough Transform technique then transforms the filtered points into a horizontal line, as shown in Figure 13b.

### 2.6. Removal of the Ground

**Figure 14:**
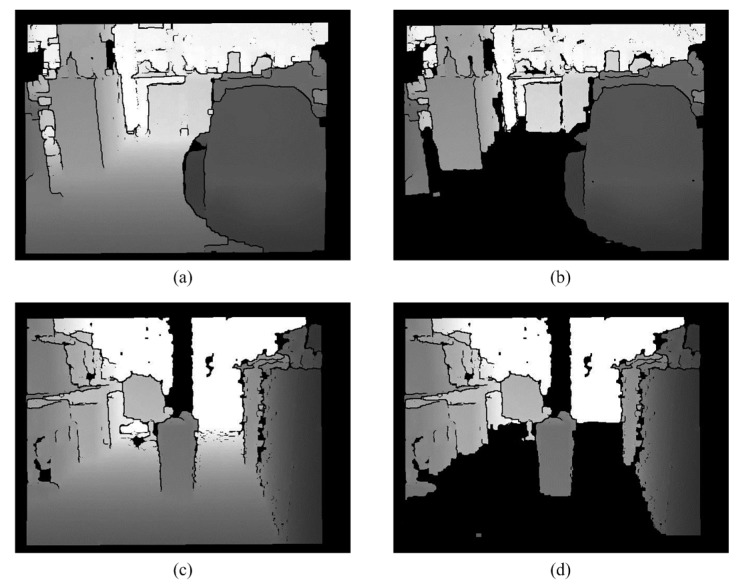
Removal of the ground. (**a**) Edge removed image 1; (**b**) Processing result of (**a**); (**c**) Edge removed image 2; (**d**) Processing result of (**c**).

**Figure 15:**
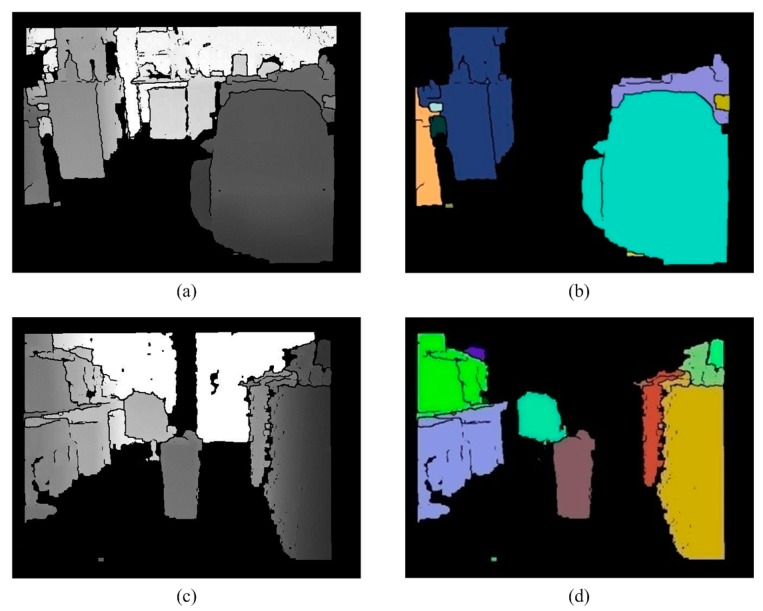
Labeling. (**a**) Ground removed image; (**b**) Labeling result; (**c**) Ground removed image; (**d**) Labeling result.

If connected component labeling or other labeling methods are directly used to label tags, it is difficult to separate the obstacles from the ground, because the junctions between the ground and the obstacles have the same depth value. Therefore, the information for the ground must be removed. RANSAC plane fitting [35,37] is used to determine the ground plane in the 3D space. Because the sensor cannot be fixed, the calculation of the ground information requires an iterative approach. In order to improve the speed of the system, [38] and the following information are used to filter out the ground: (1) The ground is usually relatively flat and (2) Using the information on depth, the gray value varies from large to small (from far to near). (3) Only the large areas of the ground are required, so Equation (5) is used. Using these features, the planes of interest meet three conditions. The regions and the sizes of the different planes of interest are determined and then the ground plane is removed using Equation (5), which has a large area. The processing result is shown in Figure 14. These separated objects are label as different color in Figure 15. The least squares method (LSM) in a quadratic polynomial is used to approximate the ground curves and to determine the ground height threshold in the V-disparity:

(5) $$Ground(x,y)=\left\{ \begin{array}{l} 0\;,\;P(x,y)-P(x,y-n)\geq 2\\ \;\wedge \overset{n-1}{\underset{i=0}{\cap }}P(x,y-i)-P(x,y-i-1)\geq 0\\ \;\wedge P(x,y)>TH1\\ unchanged\;,others \end{array}\right.$$

where P(⋅) and Ground(⋅) represent the pixel value of the coordinates (x,y), n determines the range (*n* = 10) and TH1 represents the threshold (TH1=35). These characteristics of ground plane in depth map must meet the following three points: (1) Depth values of horizontally adjacent points of ground plane are almost the same; (2) Depth values of vertically adjacent points of ground plane are gradient; (3) Depth values must be greater than *TH*1.

### 2.7. Labeling

The reason of using the labeling is easy to observe the experiment. After observations, we can stop this function, and then the performance is better. The Connected Component Method (CCM) and the region growth method [13,41] are the most common methods of labeling. The connected component method is used for a 2-D binary image. It scans an image, pixel-by-pixel (from top to bottom and left to right), in order to identify connected pixel regions, *i.e.*, regions of adjacent pixels, that share the same set of intensity values. CCM can be either 4-Connected Component or 8-Connected Component for two dimensions. The Connected Component Method can be a 6-connected neighborhood, an 18-connected neighborhood, or a 26-connected neighborhood for three dimensions. The disadvantage of the connected component method is that it is time-consuming.

A Region Growth Algorithm (RGA) is a simple, region-based image segmentation method. RGA is suitable for a gradient image. A Seeded Region Growth Method (SRG) [42] is a type of RGA. SRG is rapid, robust and allows free tuning of a parameter. SRG is faster than CCM, but it allows over-segmentation there is a problem with the initial positions of seeds. We briefly conclude the advantages and disadvantages of region growing. The advantages of region growing are as follows: (1) Region growing methods can correctly separate the regions that have the same properties we define; (2) Region growing methods can provide the original images, which have clear edges the good segmentation results; (3) The concept is simple. We only need a small numbers of seed point to represent the property we want, then grow the region; (4) We can determine the seed point and the criteria we want to make; (5) We can choose the multiple criteria at the same time; (6) It performs well with respect to noise. The Disadvantage of region growing as following: Noise or variation of intensity may result in holes or over-segmentation. We proposed system could solve this disadvantage of region-growing techniques.

The sensing range of Kinect is 0.8 to 4.0 m. When the range is greater than the maximum distance, it cannot determine the distance, so the distant information must be removed. In order to measure distances accurately, the distance information for less than 3 m is retained.

Different tags are then placed on different objects. The general labeling methods use eight connected component labeling and region growth, but tag harmonization for connected component labeling requires much iteration, because of the complex shape of the connected area:

(6) $$S(i,j)=\left\{ \begin{array}{l} \left(i,j),\;if\;[\;(P(i-1,\;j-1)=0\right)\\ \text{ Λ}(P(i,j-1)=0)\\ \text{ Λ}(P(i+1,j-1)=0)\\ \text{ Λ}(P(i-1,j)=0)\\ \text{ Λ}(P(i,j)\neq 0)]\\ not\;seed,\;others \end{array}\right.$$

Equation (6) is 8-connnected of image processing. According to neighbor state of P(i,j), to determine P(i,j) belongs to which seed (classification). Here, S(i.j) represents the seed coordinate and P(i,j) represents the pixel value at the coordinate (i,j).

**Figure 16:**
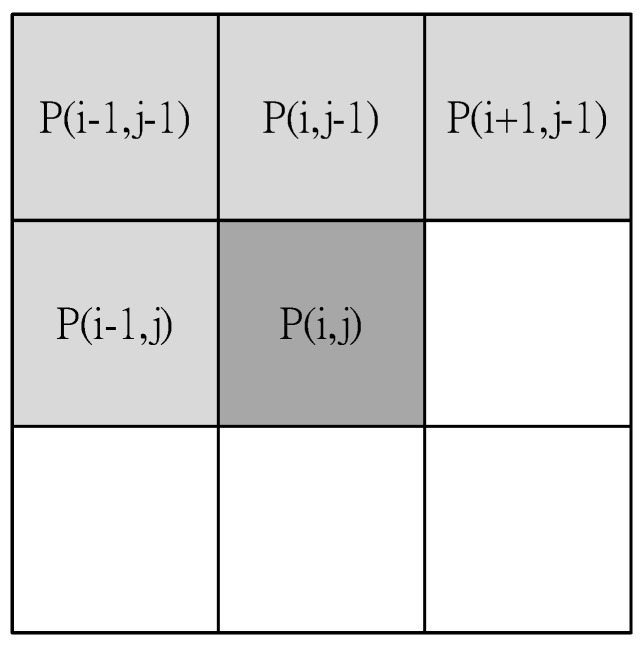
The mask for the initial seed.

**Figure 17:**
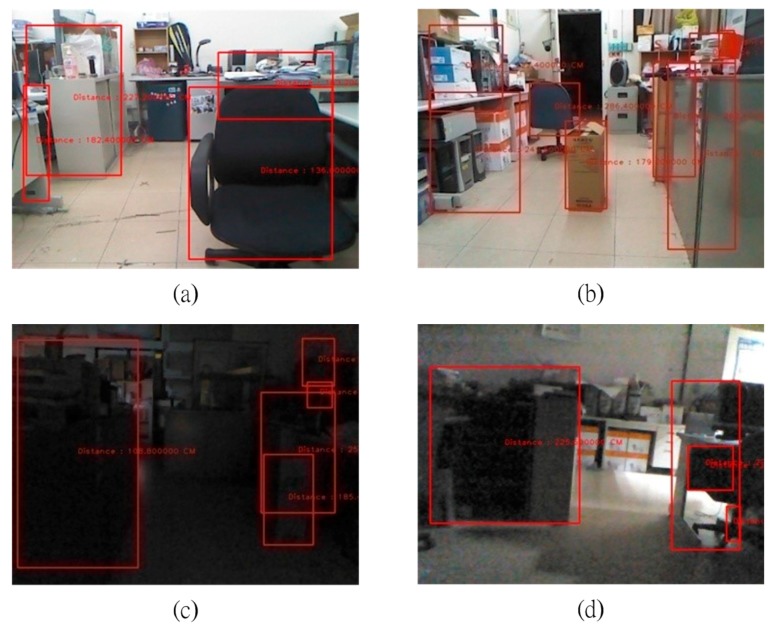
The results for obstacle detection. (**a**) Bright indoor; (**b**) Bright indoor; (**c**) Low-light indoor; (**d**) Low-light indoor.

In order to increase the efficiency of the system, Connected Component Region Growth is used. Traditional region growth initially sprinkles some seeds in the image. If the distribution of the sprinkled seeds is not appropriate, the growth results are imperfect, so the choice of the initial position of the seeds is improved in the proposed system. Information about object edges is used. Because the previous step removes the edge information for an object, each object is isolated by black color. Equation (6) and the mask for the initial seed are used to select the coordinates of initial seeds, as shown in Figure 16. These coordinates are then used to execute region growth. This ensures that each object has an initial seed and that any growth is not been repeated. Therefore, a system to reduce the amount of computation is proposed. The processing result is shown in Figure 17.

### 2.8. The Detection of Rising Stairs

**Figure 18:**
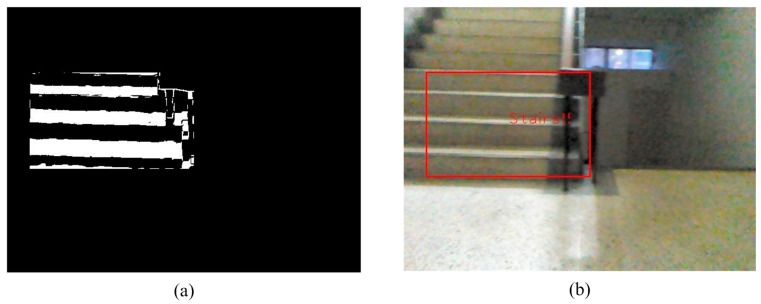
The detection of rising stairs. (**a**) Satisfied conditions of a suspicious plane; (**b**) Upstairs detection image.

The system then analyzes each of the tagged objects individually, to determine whether the object is rising stairs because of a change in depth. The rising stairs depth value has a hierarchical characteristic, from top to bottom and from large to small. When the obstacle fulfills these characteristics, it is determined to be rising stairs. The detection results are shown in Figure 18.

### 2.9. The Labeling of Objects and Informing the User

**Figure 19:**
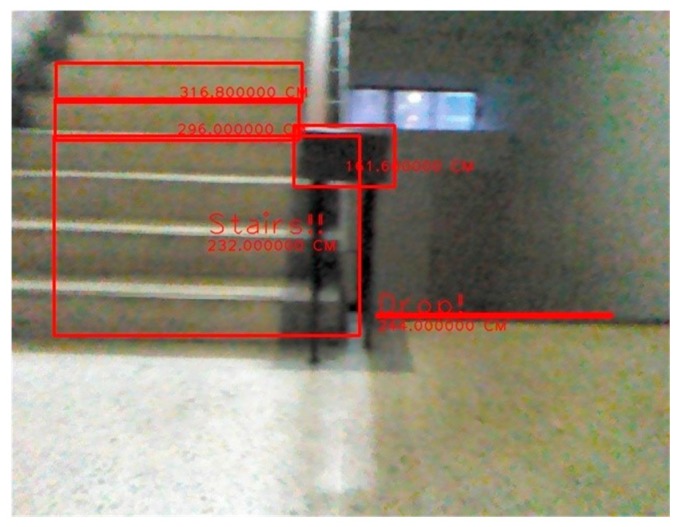
The result of labeling.

This system labels objects with rectangle. It shows the information about detected objects on the image and the distance of the obstacle or the staircase. The results are shown in Figure 19.

Finally, the system uses Text-To-Speech (TTS) software [43]. When the obstacle is in front of the user, the system vocally informs the user of the distance to the obstacle and the obstacle category. When the system detects stairs, it gives the direction and the distance to the stairs to the user to ensure the user’s safety. This vocal alarm is very short and focuses on concise information about the closest obstacle.

## 3. Experimental Results

A Microsoft Kinect sensor is a tool that captures images, as shown in Figure 5 and Table 1. The experimental platform is Windows 7. The programming language is Visual C++ 2010 with Opens 2.3, running on a notebook with an Intel(R) Core(TM) i5-3210M CPU@2.5GHz 8G 64 bits. The image resolution is 640 × 480 and the depth map capture rate is 30 frames per second. The sensing range is 0.8 to 4.0 m.

A Kinect sensor uses structured light methods to give an accurate depth map of a scene. Both the video and depth sensor cameras in the Kinect sensor have a 640 × 480-pixel resolution and run at 30 FPS (frames per second). There are two cameras and an IR projector. One camera is for color video and the other one with the IR Projector is for the depth map. Currently, there are two categories of SDK for Kinect: Open NI and Microsoft Kinect for Windows SDK.

**Figure 20:**
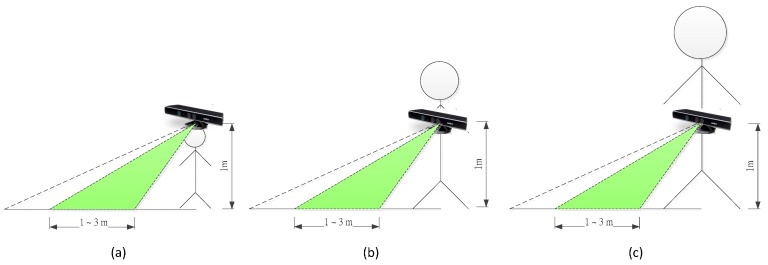
The Schematic diagram of the Kinect configuration for different stature person. (**a**) A short person; (**b**) A medium stature person; (**c**) A tall person.

Kinect configuration height and distance accuracy are related. If possible, the Kinect sensor keeps horizontally that experiment results are better. The Kinect sensor configuration is as shown in Figure 20. Our Kinect sensor is totally fixed on a helmet or chest and waist.

Infrared rays are easily affected by sunlight [44]. The Kinect sensor depends on emitted infrared rays to generate a depth map, so the Kinect sensor has some hardware limitations. The Kinect sensor is easily affected by sunlight, so it can only be used for environments that lack sunlight, such as a night scene, a cloudy day or indoors. It is worthy of note that the Kinect sensor is not totally useless outdoors, but it cannot be used in sunny environments.

In this section, all of the experiment images are random images taken from the experiment. The experiments are divided into two different environments: simple and complicated. A simple environment does not include stairs and a complicated environment has stairs. Both environments are situated indoors and outdoors, with sufficient and insufficient light. The experiments use different brightness values for the indoor and outdoor environments and for with stairs and without stairs. Figure 17a,b shows the results for a bright indoor environment. Figure 17c,d shows the results for a low-light indoor environment. When obstacles are in front of the user, the system vocally informs the user of the distance to the obstacle.

### 3.1. System Testing in a Simple Environment

This section details the success rate for obstacle detection in a simple environment without stairs. In this study, an object that affects the path of a user is defined as an obstacle. If an obstacle is labeled, the detection is successful. If not, there is a failure to detect.

### 3.2. An Indoor Environment under Sufficient Light

**Figure 21:**
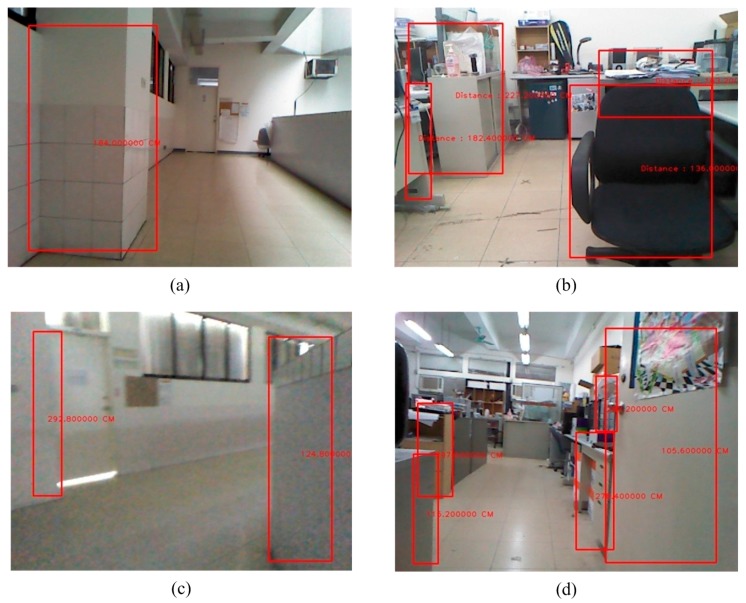
The detection of an obstacle indoors under sufficient light. (**a**) Corridor 1; (**b**) Laboratory 1; (**c**) Corridor 2; (**d**) Laboratory 2.

The detection success rate and the failure rate are shown in Table 1. As shown in Figure 21, indoor ground is flatter than outdoor ground so the projection distribution of the ground in V-disparity is more concentrated. The success rate is excellent when the ground in the depth map is removed using the ground height threshold in the V-disparity. There are some failures due to the material nature of objects, such as a large expanse of transparent glass or smooth metal.

**Table 1:** The success rate and the failure rate for the detection of obstacles.

	Frame Amount (Total 2265 frames)	Percentage (%)
Success	2201	97.17%
Failure	64	2.83%

### 3.3. An Indoor Environment under Insufficient Light

**Figure 22:**
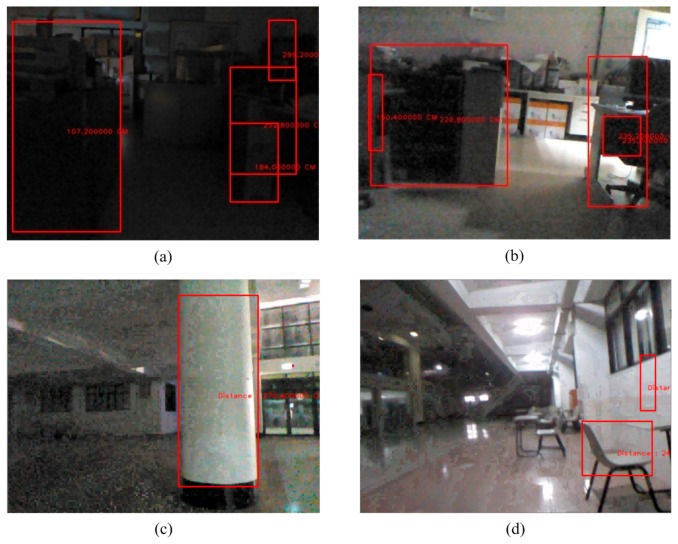
The detection of an indoor obstacle under insufficient light. (**a**) Laboratory 1; (**b**) Laboratory 2; (**c**) Lobby; (**d**) Corridor.

The detection success rate and the failure rate for obstacle detection are shown in Table 2. As shown in Figure 22, the depth information is not affected by illumination because it is obtained from the Kinect sensor. Indoor ground is flatter than outdoor ground so the projection distribution of the ground in V-disparity is more concentrated. The success rate is excellent when the ground in depth map is removed using the ground height threshold in the V-disparity. The nature of the material of an object in the scene influences the success rate, for example, glass or metal.

**Table 2:** The success rate and the failure rate for obstacle detection.

	Frame Amount (Total 213 frames)	Percentage (%)
Success	206	96.71%
Failure	7	3.29%

### 3.4. System Testing in a Complicated ENVIRONMENT

**Figure 23:**
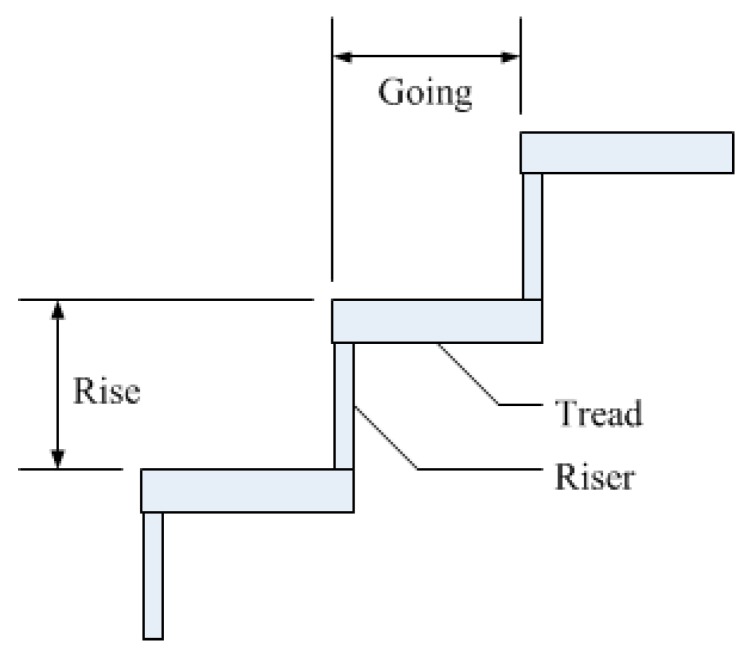
The structure of the stair.

If the test environment contains stairs, it is defined as a complicated environment. The basic structure of the stairs is shown in Figure 23. This study focuses on rising and descending stair structures. If the system identifies the obstacles and the stairs accurately, it is a successful detection. If not, then it is a failure.

### 3.5. An Indoor Environment under Sufficient Light

**Figure 24:**
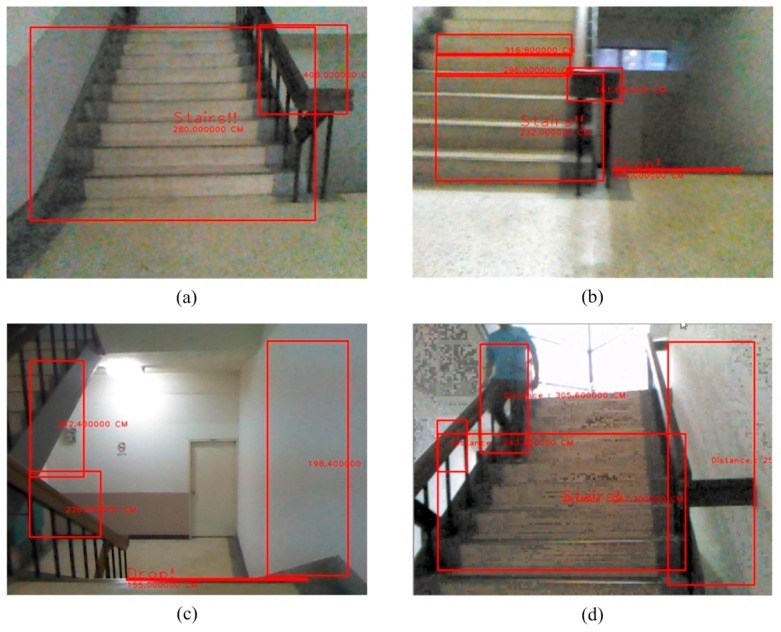
The detection of an obstacle indoors under sufficient light. (**a**) Rising stairs; (**b**) Rising and descending stairs; (**c**) Obstacle and descending stairs; (**d**) Obstacle and descending stairs.

The success rate and the failure rate for detection are shown in Table 3. The types of stairs are simpler in the indoor environment, so there is no problem with detection. Figure 24 shows that if the most of the stair structures are not obscured by person or objects, it is successfully detected. The experimental results show that as long as most of the stair is not occluded, it is successfully detected.

**Table 3:** The success rate and the failure rate for obstacle detection.

	Frame Amount (Total 262 frames)	Percentage (%)
Success	245	93.5%
Failure	17	6.5%

### 3.6. An Indoor Environment under Insufficient Light

**Figure 25:**
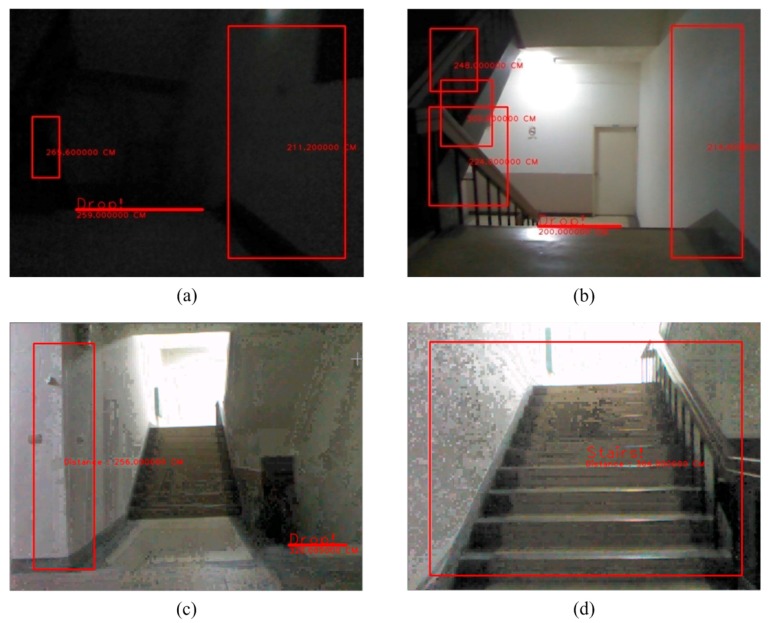
The detection of an obstacle indoors under insufficient light. (**a**) Descending stairs 1; (**b**) Descending stairs 2; (**c**) Rising stairs 1; (**d**) Rising stairs 2.

The success rate and the failure rate for obstacle detection are shown in Table 4. The success rate and failure rate for detection of descending stairs are shown in Table 5. To improve the accuracy and the capturing of images, the system uses a Kinect sensor, so that stairs can be easily detected, even in dimly lit environments as shown in Figure 25.

**Table 4:** The success rate and the failure rate for obstacle detection.

	Frame Amount (Total 104 frames)	Percentage (%)
Success	96	92.3%
Failure	8	7.7%

**Table 5:** The success rate and failure rate for detection of descending stairs.

	Frame Amount (Total 592 frames)	Percentage (%)
Success	498	84.12%
Failure	94	15.88%

### 3.7. The Confusion Matrix for Experiment Results

The indoor experimental data is expressed using a confusion matrix, as shown in Table 6. If there is a large size break in the depth map, the obstacle is not detected. When the remaining part in depth map is calculated, it is so small as to be negligible. When rising stairs are to be detected, because there are broken parts in the image depth, some blocks are mistaken for obstacles. In an indoor environment there are fewer false assessments because the ground is uniform. The probability of a false assessment is greater in an outdoor environment because the ground is diverse, such as where there is a rough surface. The detection rate for an indoor obstacle reaches 97.40%.

**Table 6:** The confusion matrix for the indoor experiment results.

Confusion Matrix		Actual Output				
		Obstacle	Upstairs	Downstairs	Barrier Free	Recognition Rate
Expected output	Obstacle	1660	0	0	24	98.57%
	Upstairs	30	382	0	0	92.72%
	Downstairs	0	0	248	13	95.02%
	Barrier free	8	0	0	524	98.50%
Accuracy rate		2814/2889				97.40%

### 3.8. The Detection of Static and Dynamic Obstacles

**Figure 26:**
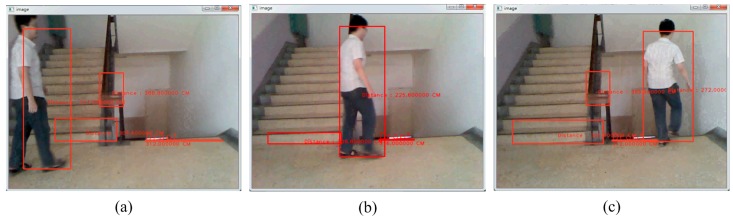
The detection of static and dynamic obstacles. (**a**) Walking people walks from the left side; (**b**) Walking people at the middle; (**c**) Walking people walks to the right side.

Our system detects static and dynamic obstacles simultaneously as shown in Figure 24d. Figure 24a–c shows static obstacle detection. As illustrated in Figure 26, this testing is for dynamic obstacle detection. The scenario is that one man walks from the left to the right in the scene.

### 3.9. The Evaluation of the System by Blind and Blindfolded Participants

**Figure 27:**
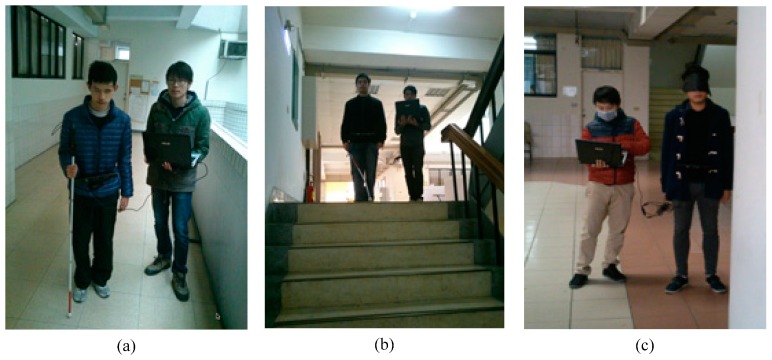
Blind and blindfolded participants. (**a**) Blind participant 1; (**b**) Blind participant 2; (**c**) Blind-folded participant.

Three blind university students (as shown in Figure 27a,b) and thirty-eight blindfolded university students were used to evaluate the system. The system is not meant to take the place of a cane or a guide dog but to improve perception using a depth sensor-based sound system. A traditional cane, which is the standard navigation tool for the blind, is difficult to replace because a cane is cheap, light and can be folded.

**Figure 28:**
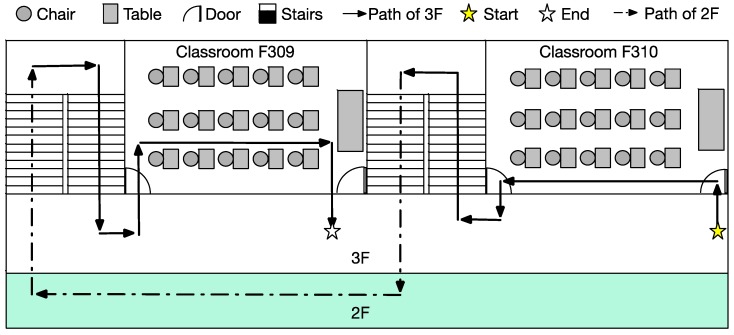
The experimental environment.

**Figure 29:**
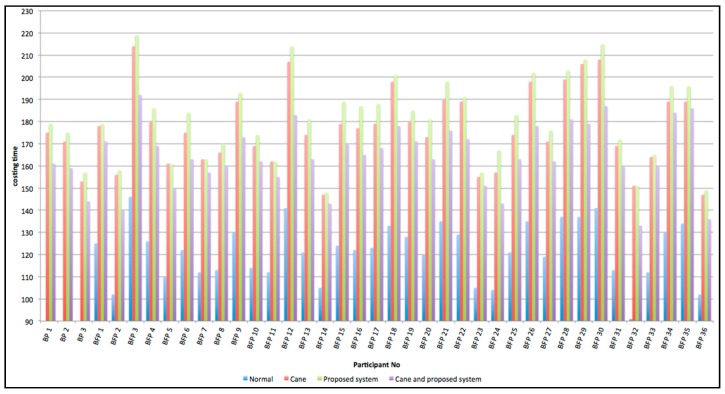
The statistical data of experiment.

**Figure 30:**
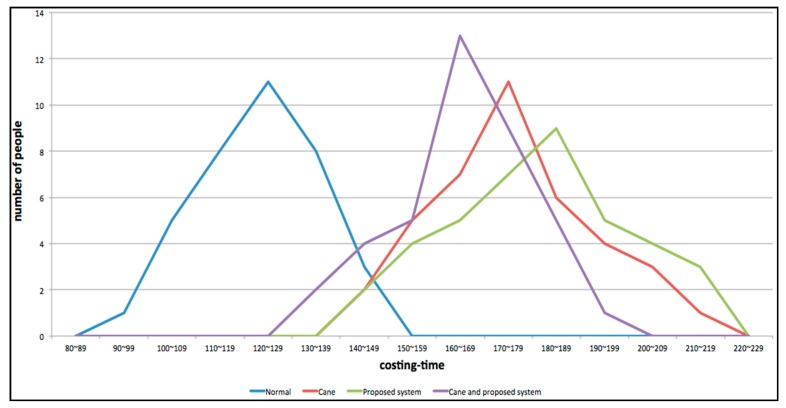
The distribution of the experimental data.

These experiments use a control experiment. There is an experimental group and a control group. The experimental environment (as shown in Figure 28) includes rising stairs, descending stairs, static obstacles and dynamic obstacles along a specific path. The participants consisted of three blind junior students (Blind Participants: BP) and thirty-eight junior students (Blindfolded Participants: BFP). The best and worst experimental results were removed. The distribution of the experimental data is shown in Figure 29. Figure 30 shows that experimental results when only the proposed system is used are similar to the experimental results when only a cane is used. However, using the system and a cane together gives significantly improved experimental results that are closer experimental results of normal people.

We calculate the *p*-value for the cane and proposed system with cane as shown in Table 7. The calculating result of *p*-value is 0.001508556 (two-tail). In general, the significance level is 0.05 or 0.01. In our case, the two-tailed *p*-value suggests rejecting the null hypothesis of no difference. The *p*-value is less than 0.5 or 0.01, so the result is significant improvement.

**Table 7:** *t*-Test: Paired Two Sample for Means.

	Cane	Cane and Proposed System
Mean	176.4615385	164.3846154
Variance	310.097166	213.5587045
Observations	39	39
Pearson Correlation	0	
Hypothesized Mean Difference	74	
t Stat	3.295836168	
P (T ≤ t) one-tail	0.000754278	
T Critical: one-tail	1.665706893	
P (T ≤ t) two-tail	0.001508556	
T Critical: two-tail	1.992543495	

## 4. Conclusions

This paper proposes an obstacle detection method that uses depth information. Because the depth information is obtained using an infrared sensor, the depth information is not affected by the degree of illumination. The proposed system is effective in detecting obstacles in a low light environment. The system addresses the problem of over-segmentation by removing the edge and eliminating the problem of the initial seed position for the region growth method, using CCM. It can also detect static and dynamic obstacles. These experimental results show that when only the proposed system is used similar to the experimental results when only a cane is used. However, using the system and a cane together gives significantly improved experimental results that are closer experimental results of normal people. The system is simple, robust and efficient.

Three thresholds are used: TH1=35 for the removal of the ground plane, TH2=15 for the removal of the obstacle edge and TH3=50 for the detection of descending stairs. The detection rate for an indoor obstacle is as high as 97.40%. The experimental results show that the proposed system is very robust, efficient and convenient in an indoor environment. The system can also detect rising stairs and descending stairs and ensures that visually impaired people have the environmental information that is required to avoid danger.

The system vocally informs the user of the distance of an obstacle and the category of the obstacle. This voice alarm is very short and focuses on the most concise information about the closest obstacle. The TTS voice is not a natural voice so it has a robotic sound. In the future, the system will be improved to support multiple languages. Image processing performance of our proposed system for ROI or fully image is different, but they are small and almost the same. The most of calculations are based on Kinect. To detect object in fully image is easier than in ROI. Our system detects complete object, not just a part.
